# The minimal important difference of the hospital anxiety and depression scale in patients with chronic obstructive pulmonary disease

**DOI:** 10.1186/1477-7525-6-46

**Published:** 2008-07-02

**Authors:** Milo A Puhan, Martin Frey, Stefan Büchi, Holger J Schünemann

**Affiliations:** 1 Horten Centre for patient-oriented research, University Hospital of Zurich, Switzerland; 2 Klinik Barmelweid, Barmelweid, Switzerland; 3 Department of Psychiatry, University Hospital of Zurich, Switzerland; 4 Clarity Research Group, Department of Clinical Epidemiology and Biostatistics, McMaster University, Hamilton, Ontario, Canada; 5 Department of Epidemiology, Italian National Cancer Institute Regina Elena, Rome, Italy

## Abstract

**Background:**

Interpretation of the Hospital Anxiety and Depression Scale (HADS), commonly used to assess anxiety and depression in COPD patients, is unclear. Since its minimal important difference has never been established, our aim was to determine it using several approaches.

**Methods:**

88 COPD patients with FEV_1 _≤ 50% predicted completed the HADS and other patient-important outcome measures before and after an inpatient respiratory rehabilitation. For the anchor-based approach we determined the correlation between the HADS and the anchors that have an established minimal important difference (Chronic Respiratory Questionnaire [CRQ] and Feeling Thermometer). If correlations were ≥ 0.5 we performed linear regression analyses to predict the minimal important difference from the anchors. As distribution-based approach we used the Effect Size approach.

**Results:**

Based on CRQ emotional function and mastery domain as well as on total scores, the minimal important difference was 1.41 (95% CI 1.18–1.63) and 1.57 (1.37–1.76) for the HADS anxiety score and 1.68 (1.48–1.87) and 1.60 (1.38–1.82) for the HADS total score. Correlations of the HADS depression score and CRQ domain and Feeling Thermometer scores were < 0.5. Based on the Effect Size approach the MID of the HADS anxiety and depression score was 1.32 and 1.40, respectively.

**Conclusion:**

The minimal important difference of the HADS is around 1.5 in COPD patients corresponding to a change from baseline of around 20%. It can be used for the planning and interpretation of trials.

## Background

Depression and anxiety are highly prevalent in patients with chronic obstructive pulmonary disease (COPD). [1-3] There is general agreement that this common co-morbidity should be treated in order to improve patients' health-related quality of life (HRQL) but also to lower health care consumption [4-6] A number of treatment options are available such as cognitive behavioral therapies[7], antidepressants[8] or physical exercise[9] and there is an increasing number of randomised trials investigating these treatments.

The Hospital Anxiety and Depression Scale (HADS) is a widely used instrument to assess symptoms of depression and anxiety. It is not a tool to diagnose mood disorders but it has proofed to be a reliable, valid and responsive instrument to assess the severity of symptoms of mood disorders.[10] The self-administration and short completion time makes the HADS an attractive instrument for use in trials. It is, however, difficult to interpret treatment effects because the minimal important difference of the HADS it is not known[11]

The concept of the minimal important difference the smallest difference in the outcome of interest that informed patients or their proxies perceive as important and that may lead to a change in the management[12], has become the standard approach to interpret the clinical relevance of treatment effects[13,14] For example, the minimal important difference of the Chronic Respiratory Questionnaire (CRQ) has been established to be 0.5 points on the Likert-type scale from 1 to 7[15] Meta-analyses of randomised trials on respiratory rehabilitation show treatment effects between 0.5 and 1.0 on the CRQ thus exceeding the minimal important difference of 0.5 points and providing a patient-important benefit for a majority of patients.[16]

In order to understand how to interpret HADS scores we conducted an analysis to establish the minimal important difference of the HADS in COPD patients. Since a single approach is not sufficient we used anchor- and distribution-based methods to determine the minimal important difference of the HADS.[17]

## Methods

### Study and patients

We used the data of a randomized trial that compared different exercise modalities during an inpatient rehabilitation[18] COPD patients with a FEV1 ≤ 50% predicted (stage III-IV according to the Global Initiative for Chronic Obstructive Lung Disease criteria) and German as first or daily language followed an inpatient respiratory rehabilitation with a duration of approximately 3 weeks that included a median number of 13 exercise sessions and that was followed by individually prescribed home-based exercise (median number of total exercise sessions of 22 following after five weeks). The rehabilitation program also included patient education, breathing therapies and optimisation of medical therapy. We excluded patients with cardiovascular, musculoskeletal or neurological disorders only if physical exercise was not possible due to these co-morbidities. The study took place in a public rehabilitation clinic in Switzerland (Klinik Barmelweid, Aargau). The responsible ethics committee approved the study protocol and all study participants provided written informed consent.

### HADS

Patients completed the self-administered and validated German version of the HADS[19] The HADS measures depression and generalised anxiety in in- and outpatients and in community settings. It contains 14 statements describing symptoms of depression and anxiety (for example "I feel tense and irritable"). Response options for each question range from 0 to 3 and ask patients about their agreement with the statements or how often they apply (for example "most of the time, often, from time to time or not at al"). There are seven statements for each depression and anxiety. Domain scores range from 0 (no depression or anxiety) to 21 and following the standard convention scores ≥ 11 indicate a probable clinical diagnosis of depression or anxiety.

### Patient-important outcomes used as anchors

We used the CRQ and the Feeling Thermometer as potential anchors to determine the minimal important difference of the HADS. The CRQ is a widely used instrument in respiratory rehabilitation and measures dyspnea, fatigue, emotional functioning and coping with COPD.[20] Domain and total scores are presented on a Likert-type scale from 1 (most severe impairment) to 7 (no impairment). We used the self-administered German version[21] with standardized dyspnea questions[15] The Feeling Thermometer is a validated preference-based instrument with marked intervals from 0 (worst health state = dead) to 100 (perfect health) and it is increasingly used as a global estimate of the effect of interventions, including respiratory rehabilitation[22,23]

### Statistical analysis

For the anchor based approach we followed the within-patient anchor based method.[23] With this approach the minimal important difference of the instrument of interest (HADS) is estimated based on anchors (CRQ and Feeling Thermometer) for which the minimal important difference has been established before. An equation is derived based on linear regression analysis where the instrument of interest is the dependent and the anchors the independent variable. Using the equation one can estimate the minimal important difference of the instrument of interest.

In our analysis, we first assessed the correlation between the anchors (CRQ and Feeling Thermometer) and the HADS domain and total score. We decided to use linear regression analyses with HADS domain and total scores as the dependent and the anchors as independent variables if correlation coefficients exceeded 0.5.[23] Using the regression equation and the minimal important difference of the anchors (0.5 points for the CRQ[15] and 8 points for the FT[23]) we estimated the minimal important difference of the HADS domain and total scores.

We used the Effect Size approach as distribution-based method based. The Effect Size approach expresses treatment effects as standard deviation (SD) units of change scores (difference between baseline and follow-up). 0.5 SD units represent a moderate effect size and investigators usually consider this estimate to correspond to the minimal important difference[24] We conducted all analyses using SPSS for Windows (version 12).

## Results

We included 88 patients with complete data in this analysis. 10 patients did not complete the HADS at the follow-up after five weeks because they did not return to the study center for the follow-up assessment or because they did not return the questionnaire by mail. They did not differ from patients included in the analyses. The mean age of included patients was 68.7 (SD 8.9) years, 59 (67.0%) were males, patients had moderate to very severe COPD with a mean FEV_1 _in % predicted was 34.3% (SD 8.2), mean years since diagnosis was 9.3 years (SD 7.3) and mean number of pack years was 52.3 (SD 28.7) years. 49 (55.7%) had suffered from an exacerbation in the previous eight weeks and 49 (55.7%) had cardiovascular co-morbidity.

The mean HADS depression score at baseline was 7.63 (SD 3.9) and 19 (21.6%) patients had scores ≥ 11. For the HADS anxiety domain, mean score was 7.03 (SD 4.0) and 20 (22.7%) patients had scores ≥ 11. Table 1 shows the changes from baseline to follow-up for HADS, CRQ and Feeling Thermometer scores and the correlations between outcomes. The change scores for the CRQ and Feeling Thermometer both exceeded the threshold of their minimal important difference (0.5 and 8 points, respectively). Correlations were highest between the CRQ emotional function domain and HADS scores and lowest between the CRQ dyspnea and Feeling Thermometer and the HADS scores. We found strong correlations (≥ 0.5) between the HADS anxiety domain and the CRQ emotional function and mastery domains and between the HADS total score and the CRQ emotional function and total score. None of the correlations between the HADS depression score and anchors were ≥ 0.5.

**Table 1:** Changes^# ^in HADS and CRQ and Feeling Thermometer scores and correlations^‡ ^of changes

		**HADS depression ** **domain**	**HADS anxiety ** **domain**	**HADS total score**
	Changes from baseline to follow-up	-2.44 (2.79)	-2.02 (2.65)	-2.23 (2.34)
**CRQ dyspnea**	1.25 (1.17)	-0.24	-0.17	-0.24
**CRQ fatigue**	0.94 (1.25)	-0.43	-0.37	-0.46
**CRQ emotional function**	0.96 (1.07)	-0.42	**-0.55**	**-0.56**
**CRQ mastery**	0.94 (1.28)	-0.28	**-0.51**	-0.45
**CRQ total**	1.02 (0.99)	-0.41	-0.48	**-0.51**
**Feeling Thermometer**	11.16 (15.82)	-0.23	-0.21	-0.25

^# ^Values for changes are means (standard deviation).

^‡ ^Pearson correlation coefficients. Values in bold indicate sufficiently high correlations for using the anchor based method (linear regression analysis)

Table 2 shows the minimal important difference estimates based on the anchor-based methods. The minimal important difference estimates were consistent across the four regression models and between 1.41 (95% CI 1.18–1.63) and 1.68 (1.48–1.87). The minimal important differences were a little lower for the distribution-based method. Based on the Effect Size approach the minimal important difference was 1.40 for the HADS depression score, 1.32 for the HADS anxiety score and 1.17 for the HADS total score.

**Table 2:** Anchor-based method to determine the minimal important difference of the HADS

	**Regression equation**	**Corresponding to 0.5 change in CRQ ** score (95% confidence interval*)
			
**Change in HADS **	0.73 + 1.35*CRQemotional function, r^2 ^= 0.30	1.41 (1.18–1.63)
**anxiety score**	1.04 + 1.05*CRQmastery, r^2 ^= 0.26	1.57 (1.37–1.76)

**Change in HADS total score**	1.07 + 1.21*CRQemotional function, r^2 ^= 0.31	1.68 (1.48–1.87)
	1.00 + 1.20*CRQtotal, R^2 ^= 0.26	1.60 (1.38–1.82)

Constant and coefficients correlations multiplied by -1 to facilitate interpretation.

* The 95% confidence intervals around the minimal important difference should not be used to make treatment decisions or develop trials without the understanding that the point estimate is the best estimate of the minimal important difference and that the limits of the 95% confidence intervals are sample size dependent. Since this sample is relatively small, the 95% confidence intervals are wide and, thus, attention must be paid to this issue. The point estimate should be used as best estimate.

## Discussion

This analysis showed that the minimal important difference of the HADS is approximately 1.5 points in COPD patients. Investigators and those interpreting clinical research can use this minimal important difference to determine whether treatment effects are in a range that is important to patients and would indicate a positive effect.

A strength of this study is the use of different approaches to establish the minimal important difference as none of the single approaches is without limitations.[17] In addition, we used a rigorous criterion for the anchors (correlations had to be ≥ 0.5) because an external anchor provides a valid estimate of the minimal important difference only if the correlation between the target instrument and the anchor is sufficiently high.[17] As a consequence of correlations below 0.5, we could not use the anchor-based approach to estimate the minimal important difference of the HADS depression score. Also, the Feeling Thermometer could not be used at all. Correlations with HADS domain and total scores were surprisingly low compared with those observed in earlier studies.[22] A possible explanation for lower correlations is that inclusion criteria for randomised trials as ours are usually stricter than those of non-randomised studies, which is the common study design for validation studies. Smaller between-person differences, as a consequence of stricter inclusion criteria, may have a substantial (negative) impact on correlation coefficients.

Anchor-based methods yielded somewhat higher minimal important difference estimates than the distribution-based method. Differences between the methods do not appear to be significant as the distribution-based estimates were within 95% confidence intervals of the anchor-based estimates. A likely explanation for the lower estimates is that we used only one study. Distribution based methods tend to underestimate the minimal important difference if based on single studies because distributions are narrower or SD smaller, respectively, as a consequence of eligibility criteria. Therefore, we would welcome further analyses that, optimally, pool data from different studies in order to include a population that is as broad as possible.

Awareness that anxiety and depression are common co-morbidities in chronic disease has risen over the last decade [1-3] But recent systematic reviews of common treatments such as cognitive behavioral therapies[7], antidepressants[8] or physical exercise[9] show that evidence is still scarce. Few trials on physical exercise used, for example, an instrument for symptoms of depression or anxiety. Only one large trial.[25] used the HADS so far. It found, after six weeks of rehabilitation in patients with COPD, reductions of 1.3 points (95% CI 0.6–2.4) for anxiety and 2.1 points (95% CI 1.3–2.8) for depression scores. Thus for anxiety, the effect might just be of borderline importance to patients whereas the majority of patients perceived a benefit for depressive symptoms. For any treatment of depression and anxiety in diseases such as COPD evidence is still lacking to provide strong recommendations. However, the treatment of depression in disease such as COPD will be increasingly important. In trials using the HADS the MID estimate of 1.5 points will play an important role to interpret treatment effects.

The minimal important difference also plays an important role to determine sample sizes of trials. It provides the ideal base for specifying the patient-important difference that investigators want to detect. To find a difference of 1.5 points at a significance level of 0.05 and with a power of 80% and assuming a SD of 4 points as observed in our study, investigators need to enroll 112 patients in each group. If a power of 90% is desired as it may be for equivalence trials, 150 patients would be needed in each group. The CIs around the minimal important difference of 1.5 should not be used to determine sample sizes of trials and to make treatment decisions without the understanding that the point estimate of 1.5 is the best estimate of the minimal important difference and that the limits of the CIs are sample size dependent. Since this sample is relatively small, the CIs relatively are wide and, thus, attention must be paid to this issue. We suggest that the point estimate of 1.5 is used as best estimate.

We do not know whether the minimal important difference of 1.5 generalizes to patients with other diseases. Patients included in our study might, however, represent patients with advanced chronic disease because mean HADS anxiety (7.03) and depression scores (7.63) were in the range commonly encountered in patients with chronic disease.[26,27] A change of 1.5 points corresponds approximately to a 20% change from these baseline scores. In patients with substantially lower or higher scores, the minimal important difference might be smaller or larger, respectively, but it would be important to know whether a 20% change would represent the minimal important difference as well. Other studies should investigate the minimal important difference of the HADS in order to interpret and plan studies outside of COPD.

## Conclusion

Our analysis shows that the minimal important of the HADS is around 1.5 points in COPD patients corresponding to a change from baseline of around 20%. This estimate is informed by both anchor- and distribution-based methods. The minimal important difference informs clinicians to interpret the importance of treatment effects on depression and anxiety in patients with COPD and provides an evidence base for sample size calculations in trials where investigators use the HADS as the primary outcome.

## Authors' contributions

MP participated in the design of the study, performed the statistical analysis and drafted the manuscript. MF participated in the design of the study, collection of the data and revised the manuscript. SB revised the manuscript. HJS participated in the design of the study, performed the statistical analysis and revised the manuscript. All authors read and approved the final manuscript.

## Conflict of interest statement

Holger Schünemann is one of the developers of the CRQ-SAS. HJS is editor in chief of HQLO and Milo Puhan Associate Editor. The article underwent regular blind peer review.
